# Metabolomics Analysis of *Morchella* sp. From Different Geographical Origins of China Using UPLC-Q-TOF-MS

**DOI:** 10.3389/fnut.2022.865531

**Published:** 2022-04-05

**Authors:** Hui Dong, Xiaoyan Zhao, Min Cai, Haotian Gu, Hengchao E, Xiaobei Li, Yanmei Zhang, Huan Lu, Changyan Zhou

**Affiliations:** ^1^Laboratory of Agro-Food Quality and Safety Risk Assessment (Shanghai), Institute of Agro-Food Quality Standard and Testing Technology, Shanghai Academy of Agricultural Sciences, Shanghai, China; ^2^Shanghai Engineering Research Center of Low-Carbon Agriculture (SERCLA), Eco-Environmental Protection Research Institute, Shanghai Academy of Agricultural Science, Shanghai, China; ^3^National Research Center of Edible Fungi Biotechnology and Engineering, Institute of Edible Fungi, Shanghai Academy of Agricultural Sciences, Shanghai, China

**Keywords:** *Morchella*, metabolomics, geographical origins, UPLC-Q-TOF-MS, quality evaluation

## Abstract

The morel mushroom (*Morchella* sp.) is reputed as one of the most highly-prized edible fungi with mounting cultivated area as well as commercial popularity in China. To date, optimized methods specific for quality evaluation and constituent analysis of *Morchella* sp. are still non-available, impeding the healthy and sustainable development of this industry. Herein, an untargeted UPLC-Q-TOF-MS-based metabolomics approach was performed to characterize the metabolite profiles of morel samples from four distinct geographical origins of China, *viz*. Gansu, Guizhou, Liaoning, and Henan province. A total of 32 significantly different metabolites assigned to lipids (19), organic acids (9), amino acids (3), and ketones (1) were identified to distinguish the geographic-segregation samples amenable to multivariate analysis. These metabolites may serve as molecular markers indicative of specific regions. More importantly, the lipid, protein and amino acid metabolism were responsible for geographic differences as revealed by KEGG pathway enrichment analysis. Collectively, this study not only pioneered high-throughput methodology to evaluate quality of *Morchella* sp. and distinguish geographical origins in a sensitive, rapid and efficient manner, but also shed light on the potential link between physiochemical variation and geological origins from a metabolic perspective, which may be conducive to the advancement of edible fungi industry and establishment of food traceability system.

## Introduction

The morel mushroom (*Morchella* sp.) from the edible ascomycetous mushroom family, is highly prized for its edibility, unique flavor as well as rich nutritional value (1). A wealth of pharmacologically active components in *Morchella* sp., including polysaccharides, terpenoids, ergosterol derivatives, microthecin, and phenolic contents have been well-documented (2–4). In some Asian countries, especially in China and India, the morels are also used as a conventional herbal medicine to cure some diseases such as hypertension, hypercholesterolemia, and cancer through the ages (5). Despite its wide geographic distribution, the morels have only been successfully domesticated in several countries, such as China and America (6). With its tremendous market popularity and economic value, the morel variety emerged as the booming mushroom industry. For instance, by the end of 2019, the total annual output of morels in China reached 72.47 thousand tons, making it the largest producer of cultivated morels around the world.

In China, the morels are dominantly distributed in the southwest, northwest and northeast areas. Morels of geographical origins may possess substantially different qualities and thus different market price, which is the major concern for consumers (7). The different qualities of morels exhibit different qualities may be attributed largely to cultivation conditions in their geographical origin. Aside from this, environmental factors, growers' preferences, national and local policy, production season, genetic traits, storage technology and/or conditions also resulted in the physiochemical variation of them (8). Thus far, there is scanty information regarding methods for the assessment of the qualities and quantification of constituents of morels from different geographical origins.

As the promising and rapidly developing technology of post-genomics era, metabolomics generates intensive interest in scientific communities over the past few years. It is the comprehensive study of the composition and dynamic changes of small-molecule metabolites (<1,000 Da) within a biological system under specific sets of environmental conditions at cell, tissue, or individual levels (9, 10) as per multiple detection methods, i.e., nuclear magnetic resonance (NMR), near-infrared spectroscopy (NIR), or mass spectrometry (MS)-based platforms (11–13). Compared with other metabolomics platforms, ultra-performance liquid chromatography coupled with electrospray ionization quadrupole-time-of-flight-mass spectrometry (UPLC-Q-TOF-MS) has a higher resolution, sensitivity and peak reproducibility, enabling the rapid relative quantitative or qualitative analysis of multiple compounds simultaneously (14, 15). Since morel is a complex matrix composed of thousands of metabolites, potentially the MS-based metabolomics approach is a powerful tool for identifying the nutritional and quality indexes of morels as well as understanding how they are affected by geographical origins nutritional values and quality. The specificity of morels cultivated in different regions in China is an important goal of this study.

In the present work, the UPLC-Q-TOF-MS metabolomics approach was adopted to examine physiochemical differences between the morel samples at the metabolite level from Gansu (northwestern China), Guizhou (southwestern China), Liaoning (northeastern China), and Henan (mid-eastern China). Our primary objective was to characterize metabolic profiles of morels from different origins and salient metabolites representative of specific area. The results can be used to evaluate the nutritional properties of morels and contribute to discriminating their geographical origins.

## Materials and Methods

### Samples and Reagents

**Figure 1:**
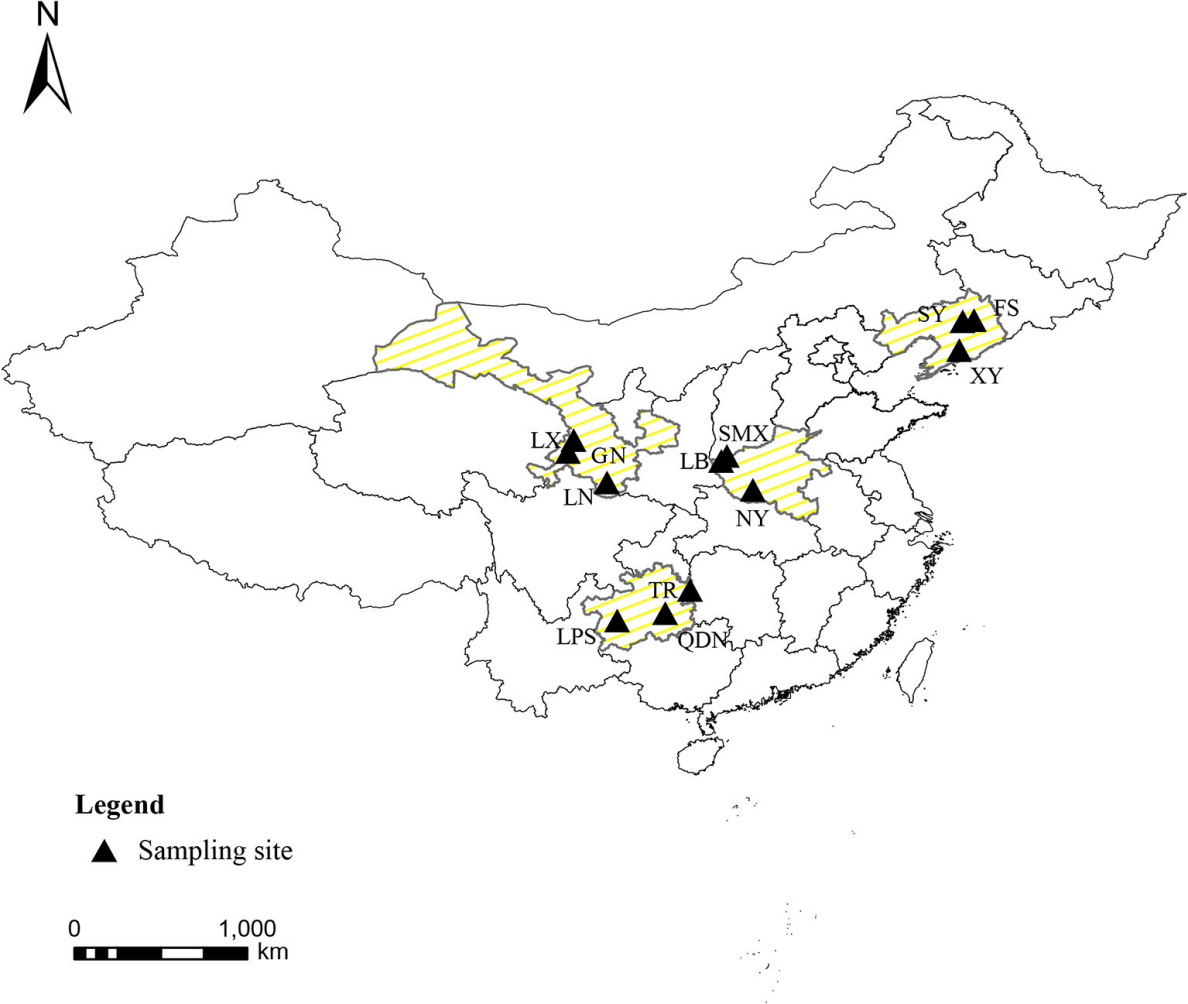
Geographical distribution of the *Morchella* sp. samples.

The cultivated fresh morels were collected from farms in four typical cultivating provinces of China in May 2018 during their fruiting period. The southwest region (Guizhou), northwest region (Gansu), northeast region (Liaoning), and eastern central region (Henan) were intentionally chosen to encompass the major production areas of morels in China, signifying the populations that were acclimatized to different topography and climate. The climatic data of the planting areas obtained from the China Meteorological Administration (http://data.cma.cn). In terms of four different regions, the average temperature throughout the entire growing season of morels were 10–18°C (Guizhou), 8–17°C (Henan), −2 to 11°C (Liaoning), and 1–12°C (Gansu), respectively. In addition, the geographical distribution is illustrated in Figure 1, and the sample information is presented in Table 1. Overall, 60 authentic morel samples were selected for analysis (*n* = 15, *N* = 4). The species of morels were identified by sequencing ITS 1 (5′-TCCGTAGG TGAACCTGCGG-3′) and ITS 4 (5′-TCCTCCGCTTATTGATATGC-3′) regions, blasted with the database of National Center for Biotechnology Information (NCBI; https://blast.ncbi.nlm.nih.gov). The 60 strains of *Morchella* sp. shared over 97% homology with each other (Supplementary Table 1). All the samples were thoroughly freeze-dried using a FreeZone freeze dryer (GOLD-SIM, USA) at −50°C under a vacuum of 1–10 mbar and stored at −20°C.

**Table 1:** The detailed information of *Morchella* sp. samples.

**Province**	**Area**	**Longitude (**°**E)**	**Latitude (**°**N)**
Gansu	GN (Gannan)	102.91	34.98
	LN (Longnan)	104.93	33.39
	LX (Linxia)	103.21	35.60
Liaoning	XY (Xiuyan)	123.28	40.29
	SY (Shenyang)	123.47	41.81
	FS (Fushun)	124.04	41.85
Henan	SMX (Sanmenxia)	111.19	34.77
	LB (Lingbao)	110.89	34.52
	NY (Nanyang)	112.54	33.00
Guizhou	QDN (Qiandongnan)	107.98	26.58
	LPS (Liupanshui)	105.48	26.20
	TR (Tongren)	109.26	27.82

Methanol and acetonitrile were purchased from ANPEL (Shanghai, China). Formic acid and ammonium acetate were obtained from Sigma-Aldrich (Madrid, Spain). Ultra-pure water (18 mΩ) was used throughout the study which was produced by Milli-Q water purification system (Millipore, Milford, MA, USA). All chemicals and solvents were analytical or HPLC grade.

### Sample Preparation

All the frozen samples were ground to a fine powder (passed through a 425 μm mesh sieve). For the UPLC-Q-TOF-MS analysis, 0.5 g of the fine powder was dissolved in 5.0 mL of 75% methanol and then ultrasonically extracted for 30 min. The extracts were then centrifuged at 12,000 rpm at 4°C for 20 min. Subsequently, the supernatants (100 μl) from each tube were filtered through 0.22 μm microfilters and transferred to LC vials. The vials were stored at 4°C until the UPLC-Q-TOF-MS analysis. In addition, the quality control (QC) sample was obtained by mixing aliquots of all samples, vortex-mixed thoroughly and transferred into the LC vial for analysis.

### UPLC-Q-TOF-MS Data Acquisition

The UPLC-Q-TOF-MS analysis was performed on an ACQUITY UPLC system (Waters Corporation, Milford, USA) coupled with an AB SCIEX Triple TOF 5600 System (AB SCIEX, Framingham, MA, USA). AN ACQUITY UPLC BEH C18 column (2.1 × 100 mm, 1.7 μm, Waters, Milford, MA, USA) was employed for sample separation in both positive and negative modes. The binary gradient elution system consisted of (A) acetonitrile and (B) water (containing 0.1% formic acid, v/v). The separation was achieved using the following gradient: 0–2 min, 5% A; 2–15 min, 5–95% A; 15–17 min, 95% A; 17–17.1 min 95–5% A; 17.1–20 min 5% A. The flow rate was 0.35 mL/min and the column temperature was 35°C. All the samples were kept at 4°C during the analysis. The injection volume was 3 μL. Data acquisition was performed in full scan mode (m/z ranges from 100 to 1,000) combined with IDA mode. Parameters of mass spectrometry were as follows: Ion source temperature, 550°C (+) and 550°C (–), ion spray voltage, 5,500 V (+) and 4,500 V (–); curtain gas of 35 PSI; collision energy, 10 eV (+) and 10 eV (–); and interface heater temperature, 550°C (+) and 600°C (–). For IDA analysis, the range of m/z was set as 50–1,000, the collision energy was 30 eV. The extracts were analyzed in auto MS/MS mode to obtain MS2 data. Three injections of the QC sample were initially done to equilibrate the column prior to the injections of the 60 samples and injected at regular intervals (every 10 samples) throughout the analytical run to provide a set of data from which repeatability can be assessed.

### UPLC-Q-TOF-MS Data Processing and Multivariate Analysis

The accurate mass data from automatic MS/MS measurements were processed using the Progenesis QI (Waters Corporation, Milford, USA) software as per parameters: Precursor tolerance of 5 ppm, fragment tolerance of 10 ppm, retention time (RT) tolerance of 0.02 min, and noise elimination level of 10. The minimum intensity was set to 15% of base peak intensity. The Excel file was obtained with three-dimension data sets including m/z, peak RT, peak intensities, and RT-m/z pairs were used as the identifier for each ion. The resulting matrix was further reduced by removing any peaks with a missing value (ion intensity = 0) in more than 60% of samples (16, 17). Finally, all potential metabolites that met these criteria were identified by using the accurate mass, MS/MS fragments spectra and isotope ratio difference in Human metabolome database (HMDB; http://www.hmdb.ca/) and Metlin database (https://metlin.scripps.edu/). Only the metabolites with MS/MS fragments score above 30 were considered as confidently identified.

The pretreated positive and negative data were merged as a data matrix subjected to multivariate data analysis. Principle component analysis (PCA) and orthogonal partial least-squares-discriminant analysis (OPLS-DA) were manipulated to visualize the metabolic alterations among experimental groups, after mean centering (Ctr) and Pareto variance (Par) Scaling, respectively. The Hotelling's T2 region, shown as an ellipse in score plots of the models, defines the 95% confidence interval of the modeled variation. Variable importance in the projection (VIP) ranks of the overall contribution of each variable to the OPLS-DA model, and those with VIP >1 are considered relevant for group discrimination. An additional criterion for the inclusion of metabolites was the *p* < 0.05 (Independent-samples *T*-test). In this study, the default 7-round cross validation was applied with 1/seventh of the samples being excluded from the mathematical model in each round, to guard against overfitting. All the above chemometrics tools were operated by R (version 3.6.3) to visualize differences among groups and to yield potential markers. The possible metabolic pathways were identified by searching the online Kyoto Encyclopedia of Genes and Genomes (KEGG) database [http://www.genome.jp/kegg/pathway.html; (18)].

## Results

### Identification of Metabolites in Morels

Supplementary Figure 1 showed total ion chromatograms (TICs) of UPLC-Q-TOF-MS acquisitions of *Morchella* sp. from the four regions. A total of 864 metabolites ions were identified from the morels and after normalization, 726 ions remained. The obtained metabolites include polysaccharide, glycoside, nucleoside, nucleotide, phenols, alcohols, acids, lipids, esters, alkaloids, vitamins, amino acids, ketones, peptides, quinones, amides, sugar phosphates, coenzyme, and energy charged compounds. Most of them were involved in important synthesis pathways including glycolysis, acid cycle, pentose phosphate, purine, and nucleoside metabolism which were pivotal for morel growth and quality.

### Non-targeted Analysis by Multivariate Statistics

**Figure 2:**
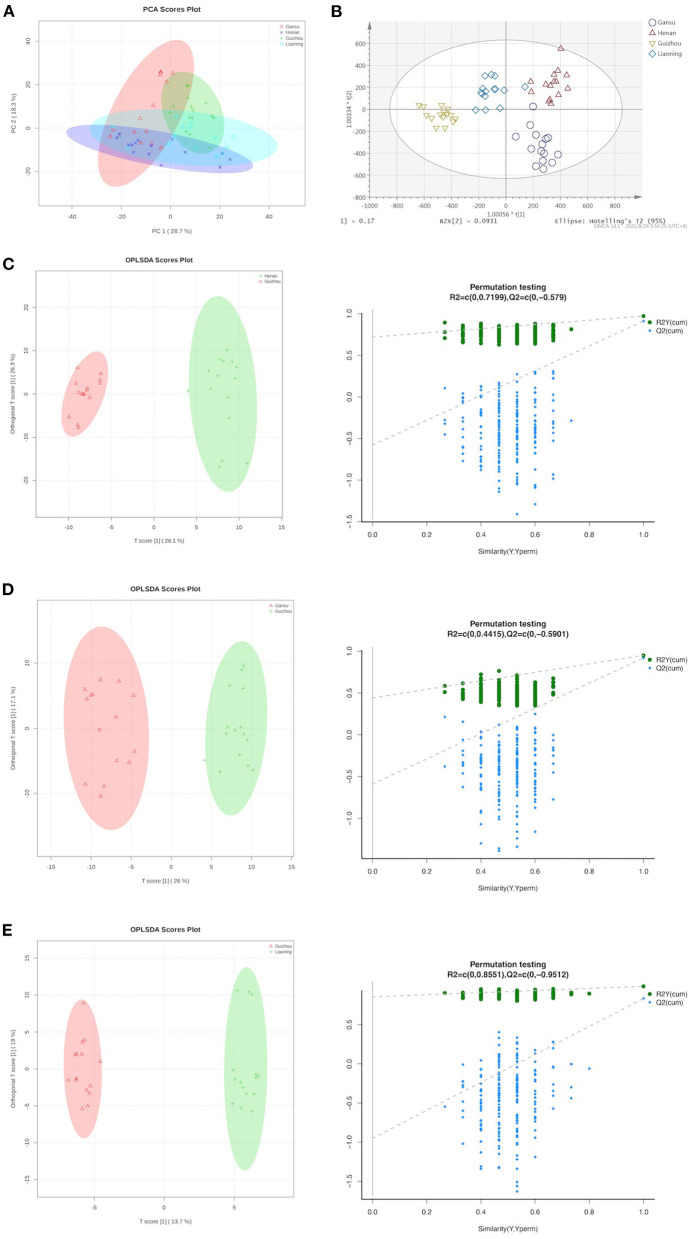
Supervised and unsupervised multivariate analysis based on metabolomic data of morels. **(A)** Score plot of principal component analysis. **(B)** Score plot of orthogonal partial least squares discriminant analysis. **(C)** OPLS-DA scores plot and permutation testing between Henan and Guizhou. **(D)** OPLS-DA scores plot and permutation testing between Gansu and Guizhou. **(E)** OPLS-DA scores plot and permutation testing between Guizhou and Liaoning.

To ascertain the interrelations between samples and their metabolites, the obtained UPLC-Q-TOF-MS dataset was subjected to further analysis. First, PCA was applied to evaluate the relationship between the data groups (including QC samples) based on their growing regions. PCA is an unsupervised learning method that reduces the dimensionality of a data set by extracting only the most important information for analysis. The PCA scores plot for all groups is shown in Figure 2A. The first and second principal components (PC1 and PC2) explained 28.7 and 18.3% of the accumulative variance contribution rate, respectively. Although the unsupervised PCA analysis resulted in weakly explained variances according to the geographical conditions, we still can observe that the morel metabolome of Henan and Liaoning regions showed the closest linkage and the morels from Guizhou showed the most distinctive profiles. Unlike the PCA model, OPLS-DA can identify the major difference in metabolic profiles among models and facilitate the identification of unique metabolites to better separate groups (19). Our OPLS-DA results indicated an appreciable discrimination of geographical origin, as shown in Figure 2B. Among them, Guizhou distinguished itself furthest against the other three origins.

Figure 2C presents the scatter score plot (R2Xcum = 0.723, R2Ycum = 0.973, Q2cum = 0.912) inferred from the inter-group comparison of the Henan and Guizhou group in OPLS-DA, suggesting good fitness and predictability of the model. For validation, the permutation test (R2 = 0.720, Q2 = −0.579) presented the same positive result as a discriminating model. Following this pattern, the statistical parameters of the other group comparisons are detailed in Figures 2D,E. All groups exhibited striking separation.

### Identification of the Differential Metabolites

**Figure 3:**
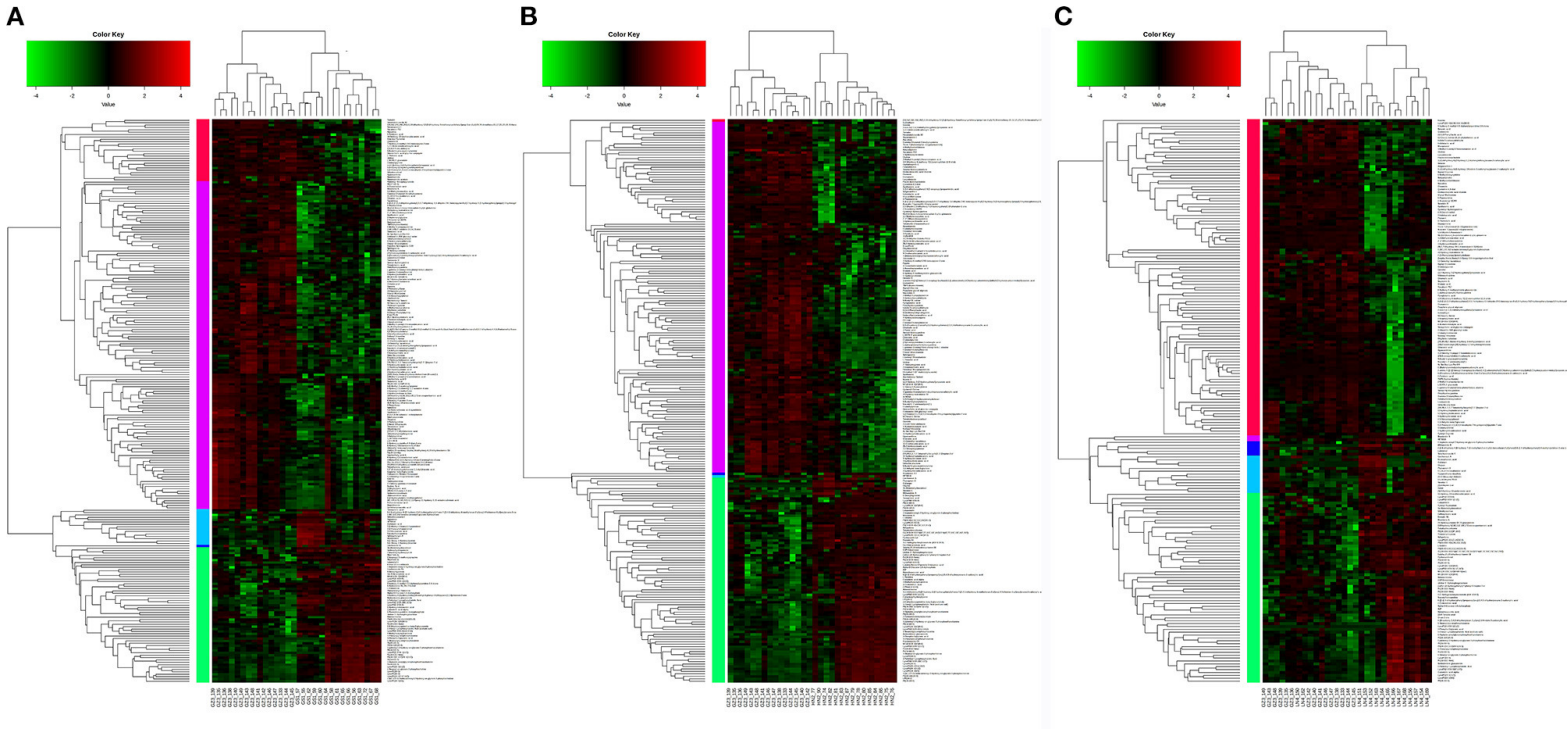
Hierarchically clustered heatmap of differential metabolites. **(A)** Gansu vs. Guizhou, **(B)** Henan vs. Guizhou, and **(C)** Liaoning vs. Guizhou.

The VIP score could be a potential indicator used to select the most discriminating geographical origin-dependent metabolites. According to the VIP >1 and *p* < 0.05, 64 significantly differential metabolites were identified between Henan and Guizhou, 66 differential metabolites between Gansu and Guizhou, and 58 differential metabolites between Liaoning and Guizhou (Supplementary Table 2). For a better visualization of the metabolites changes among different geographic origins, a cluster analysis was performed to investigate the relationships and relative abundances of the identified metabolites (Figure 3). All the morel samples from different origins showed distinct grouping patterns, consistent with the multivariate statistical analysis.

**Figure 4:**
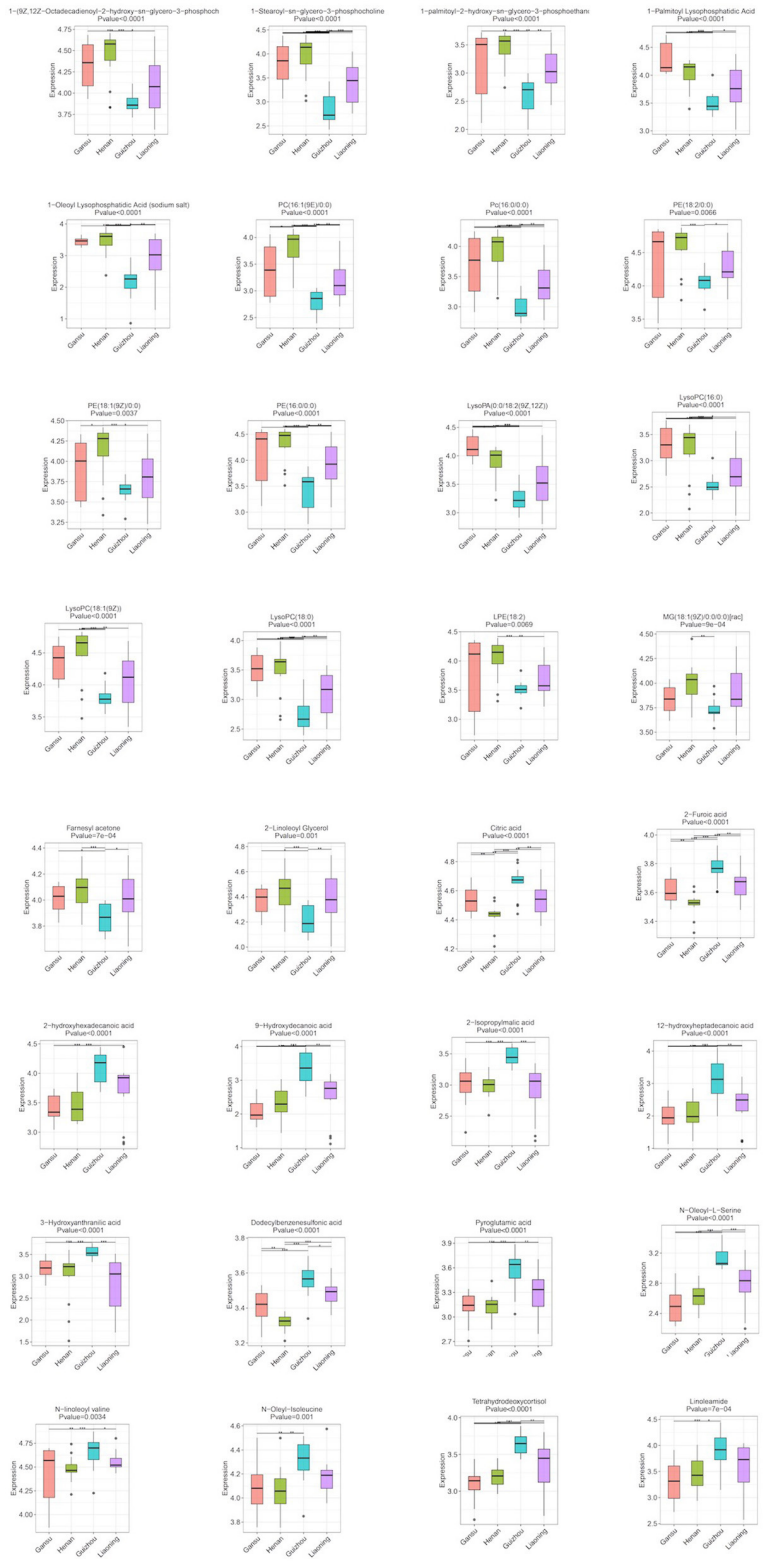
Boxplots of the normalized peak area of differential metabolites for morels samples from different geographical origins: Gansu, Henan, Guizhou, and Liaoning. Data are expressed as mean ± SD; *p < 0.05, **p < 0.01, and ***p < 0.001.

After identifying the differential metabolites with pairwise contrasts, 32 differential metabolites including 19 lipids, 9 organic acids, 3 amino acids, and 1 ketone were yielded, which can discriminate the morels of all four geographical origins and were regarded as the biomarker metabolites (Table 2). Their relative concentrations in the morel samples are shown in the corresponding loading plot (Figure 4).

**Table 2:** Differentiating metabolites between four regions with VIP > 1 and *P* < 0.05.

**No**.	**Mode**	**Metabolite**	**m/z**	**Retention time(s)**	**Formula**	**VIP**	**Fold change**	***P*-value**
**Lipids (19)**								
1	Neg	1-(9Z,12Z-Octadecadienoyl)-2-hydroxy-sn-glycero-3-phosphocholine	564.3308	7.140033	C_26_H_50_NO_7_P	5.963081	4.231249	6.35E-07
2	Pos	1-Stearoyl-sn-glycero-3-phosphocholine	524.37	10.89623	C_26_H_54_NO_7_P	3.959997	13.72693	1.34E-06
3	Pos	1-palmitoyl-2-hydroxy-sn-glycero-3-phosphoethanolamine	454.2921	7.772883	C_21_H_44_NO_7_P	2.05861	7.085745	1.59E-07
4	Neg	1-Palmitoyl Lysophosphatidic Acid	409.2358	13.68688	C_19_H_39_O_7_P	3.424535	3.439765	2.65E-06
5	Pos	1-Oleoyl Lysophosphatidic Acid	437.264	11.77503	C_21_H_41_O_7_P	2.220989	18.02467	2.32E-06
6	Pos	PC(16:1(9E)/0:0)	494.3235	6.905583	C_24_H_48_NO_7_P	3.185734	11.32803	1.34E-06
7	Pos	PC(16:0/0:0)	496.3388	7.831717	C_24_H_50_NO_7_P	3.575138	9.79105	1.61E-06
8	Neg	PE(18:2/0:0)	476.2779	7.33335	C_23_H_44_NO_7_P	6.970851	3.898707	1.95E-06
9	Neg	PE(18:1(9Z)/0:0)	478.2938	8.64645	C_23_H_46_NO_7_P	4.093172	3.623367	3.53E-06
10	Pos	PE(16:0/0:0)	454.2923	8.162367	C_21_H_44_NO_7_P	5.746396	7.895429	1.36E-07
11	Neg	LysoPA(0:0/18:2(9Z,12Z))	433.2358	13.68688	C_21_H_39_O_7_P	3.107524	4.724571	8.67E-07
12	Neg	LysoPC(16:0)	540.3305	7.833	C_24_H_50_NO_7_P	1.60656	6.473461	3.51E-05
13	Neg	LysoPC(18:1(9Z))	566.3465	8.743617	C_26_H_52_NO_7_P	6.890826	5.92695	1.43E-06
14	Neg	LysoPC(18:0)	568.3618	10.91902	C_26_H_54_NO_7_P	2.078876	6.152872	1.23E-05
15	Neg	LPE(18:2)	476.2779	7.082033	C_23_H_44_NO_7_P	3.773394	4.051591	6.45E-06
16	Pos	MG(18:1(9Z)/0:0/0:0)	357.3002	14.00458	C_21_H_40_O_4_	2.481919	1.983785	0.001477
17	Pos	2-Linoleoyl Glycerol	355.2835	12.24783	C21H38O4	3.901264	1.739285	0.000203
18	Pos	Tetrahydrodeoxycortisol	368.279	9.489133	C21H34O4	2.028486	0.363121	7E-08
19	Pos	Linoleamide	559.5183	11.37505	C18H33NO	2.481669	0.349411	0.001511
**Organic acids (9)**								
20	Neg	Citric acid	191.0198	1.072317	C_6_H_8_O_7_	5.169571	0.584352	3.56E-07
21	Neg	2-Furoic acid	111.0085	1.072317	C_5_H_4_O_3_	1.921385	0.563896	5.96E-08
22	Neg	2-hydroxyhexadecanoic acid	271.2282	12.48645	C_16_H_32_O_3_	3.497118	0.25662	3.95E-05
23	Neg	9-Hydroxydecanoic acid	187.134	5.496283	C_10_H_20_O_3_	1.719315	0.100636	0.000779
24	Neg	2-Isopropylmalic acid	175.0613	3.056717	C_7_H_12_O_5_	1.691072	0.332236	6.64E-08
25	Neg	12-hydroxyheptadecanoic acid	285.2437	12.50645	C_17_H_34_O_3_	1.474766	0.065908	0.009222
26	Neg	3-Hydroxyanthranilic acid	152.0352	3.097217	C_7_H_7_NO_3_	1.694438	0.396634	4.41E-06
27	Neg	Dodecylbenzenesulfonic acid	325.1844	15.13332	C_18_H_30_O_3_S	1.54922	0.567115	5.82E-09
28	Neg	Pyroglutamic acid	128.035	1.091483	C_5_H_7_NO_3_	1.890093	0.333157	1.21E-05
**Amino acids (3)**								
29	Neg	N-Oleoyl-L-Serine	368.2803	11.50683	C_21_H_39_NO_4_	1.234927	0.300516	1.61E-07
30	Pos	N-linoleoyl valine	380.3154	12.86563	C_23_H_41_NO_3_	4.241057	0.649084	0.001063
31	Neg	N-Oleyl-Isoleucine	394.3324	14.942	C_24_H_45_NO_3_	3.218482	0.573401	0.000871
**Ketone (1)**								
32	Pos	Farnesyl acetone	263.2365	12.24783	C18H30O	2.559786	1.706796	0.000126

### Analysis of Differential Metabolites

Lipid is a major component determining the quality of mushrooms and plants including its flavor, palatability and nutritive value (20). The multivariate analysis revealed that one of the main differences between the metabolic profiling of the four regions was related to lipids, including glycerophospholipids (15), glycerolipids (1), sterol lipid (1), and fatty acyls (2). They contributed to 19 out of 32 metabolites and showed a unique correlation that could be used to differentiate the geographical region of morels. The relative levels of these lipids revealed broadly similar trends among the four origins. All the lipids except linoleamide and tetrahydrodeoxycortisol were significantly more abundant in Henan and Gansu morels, and scarce in Guizhou. In contrast, the relative levels of linoleamide and tetrahydrodeoxycortisol showed an obviously opposite trend compared to others (Figure 3). This result indicates that the lipids metabolites might be a desirable indicator and useful for origin assessment of morel samples.

Other than lipids, multivariate analysis revealed that other 14 metabolites also played an important role in morels from different geographic origins and their differences reached a significant level, including organic acids, amino acids, and ketone. For the organic acids and amino acids, the relative levels of these 13 metabolites showed similar trends among three groups except for Guizhou where their concentrations were significantly higher. This contrasted sharply with the scenario of farnesyl acetone, since its level was significantly suppressed in Guizhou morels than others.

Considering the regulations of the 32 differential metabolites, we conducted a comprehensive analysis between different regions. PE(18:2/0:0), LysoPC(18:1(9Z)), PE(16:0/0:0), 1-(9Z,12Z-Octadecadienoyl)-2-hydroxy-sn-glycero-3-phosphocholine and citric acid had the highest contribution to the difference of these morels as indicated by their high VIP values. The morels from Guizhou had a much higher amount of Linoleamide, tetrahydrodeoxycortisol, N-Oleoyl-L-Serine, N-linoleoyl valine, N-oleyl-isoleucine, citric acid, 2-furoic acid, 2-hydroxyhexadecanoic acid, 9-hydroxydecanoic acid, 2-isopropylmalic acid, 12-hydroxyheptadecanoic acid, 3-hydroxyanthranilic acid, dodecylbenzenesulfonic acid, and pyroglutamic acid than other morels. The contents of farnesyl acetone, 2-linoleoyl glycerol and MG(18:1(9Z)/0:0/0:0) were much higher in Henan morels and Liaoning morels. While the contents of most lipids compounds such as 1-(9Z,12Z-octadecadienoyl)-2-hydroxy-sn-glycero-3-phosphocholine,1-stearoyl-sn-glycero-3-phosphocholine,1-palmitoyl-2-hydroxy-sn-glycero-3-phosphoethanolamine,1-palmitoyl lysophosphatidic acid, 1-oleoyl lysophosphatidic acid, PC(16:1(9E)/0:0), PC(16:0/0:0), PE(18:2/0:0), PE(18:1(9Z)/0:0), PE(16:0/0:0), LysoPA(0:0/18:2(9Z,12Z)), LysoPC(16:0), LysoPC(18:1(9Z)), LysoPC(18:0), and LPE(18:2) were much higher. Taken together, these 32 compounds can be used as markers to distinguish morel samples from different production regions.

Generally, the differential metabolites in Guizhou showed a particular changing tendency, which might be the result of the growing environment. Different from the plains and basins of Henan, the mountains and valleys of Gansu, the hills and plains of Liaoning, Guizhou is located on the low-latitude plateau. It has a subtropical humid climate with prominent microclimate advantages and an average relative humidity of about 80%, which is suitable for mushroom growth. Moreover, the high forest coverage rate provides a high-quality substrate source for morels production and contributes to the diverse and unique features of metabolites in morels from Guizhou.

### Analysis of Metabolic Pathways

**Figure 5:**
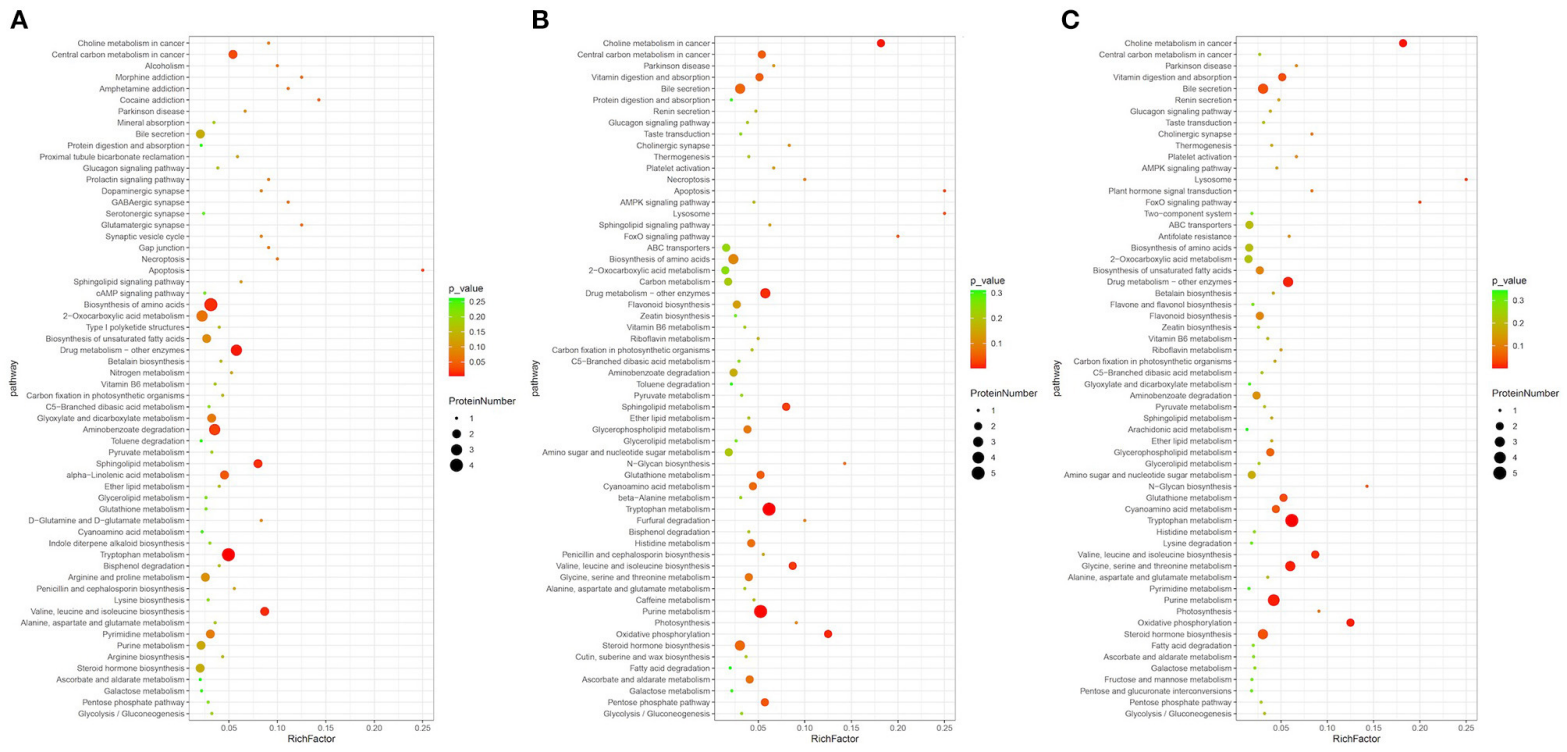
Pathway enrichment analysis of differential metabolites: **(A)** Gansu vs. Guizhou, **(B)** Henan vs. Guizhou, and **(C)** Liaoning vs. Guizhou.

The significant differential metabolites were further mapped to metabolic pathways by KEGG enrichment analysis, as shown in Figure 5. For example, in the comparison of Gansu vs. Guizhou, we found that the differential metabolites were mainly involved in tryptophan metabolism, amino acid biosynthesis and sphingolipid metabolism, which were related to the biosynthesis of proteins and lipids. Besides, other amino acid metabolism pathways were identified, such as valine, leucine, lysine, arginine, isoleucine biosynthesis, alanine, aspartate, D-glutamine, and glutamate metabolism. Generally, the lipid metabolite levels of the Henan group were elevated in these pathways while the protein metabolite levels in the Guizhou origins remained relatively stable. These results indicated that lipids, amino acids and proteins were central players in morel metabolism and their correlated pathways differed from various origins.

## Discussion

In recent years, morels have been favored by consumers for its delicious taste, excellent nutritional, and medicinal value (21). Apart from quality parameters such as sensory perception or price also geographical origins of the product strongly influence choice of consumers. To address their buying concern and portray metabolic profiles of morel from different origins, we developed a method based on untargeted metabolomics. The results suggested that morels exhibited a distinct pattern in metabolites accumulation, notably lipids, organic acids, amino acids, and its derivatives.

Lipids are mainly categorized into eight subgroups, namely fatty acyls, glycerolipids, polyketides, sphingolipids, prenol lipids, sterol lipids, saccharolipids, and glycerophospholipids (22). In our study, the main differences between the metabolic profiling of the four groups of morels were related to glycerophospholipids, glycerolipids, sterol lipids and fatty acyls. A total of 4 PCs, 4 PEs, 2 PA, 1 LysoPA, 3 LysoPCs, 1 LysoPE, 1 glycerolipid, 1 steroid, and 2 fatty acyls were differentially metabolized of morels from different geographic origins. There were significant differences in all the 19 lipid markers among the samples, and the content of lipid compounds in Guizhou is significantly different from that in the other three geographical origins, which might be caused by typically climate and different materials such as formulations of exogenous nutrition bags, although not exclusively. Tan et al. (23) found that lipids were the main carbon source of related microorganisms in the mycelium and substrate of morels. During the growth process, the contents of free fatty acids and phospholipids increased significantly, which might be caused by the hydrolysis of triglyceride stock solution or the transformation of other organic carbon nutrients. However, the carbon source consumed by morels during fruiting emergence was transferred from exogenous nutrient bags to the soil, while the decomposition ability of the carbon source was different in the process of transferring, resulting in the lipid metabolism differences in different habitats. Moreover, the composition of lipids may also be influenced by natural fluctuations in environmental conditions experienced by different geographic locations, especially the temperature (24–27). According to the literature, temperature is one of the key factors affecting the growth and development of morels. The lipid content and the unsaturation degree increased at low temperature (28). During the whole growth period, the temperature in Guizhou fluctuated slightly while for the other three geographic origins, the temperature was relatively low at the growth phases, even reaching below zero, and gradually increased when during harvesting. Consequently, the lower lipid content found in the Guizhou samples may be explained by the fact that they grew at low latitude with a warmer climate. In addition to temperature, the possible effect of other factors, such as the nutritional conditions, on the lipid content cannot be excluded.

The taste of mushroom is primarily due to the presence of many small-molecule components, including soluble sugars, polyols, amino acids, organic acids, and others (29). For the amino acids, they are important taste activating components in edible mushrooms such as alanine and glutamate with floral characteristics, and serine and tyrosine bearing wine aroma characteristics (30). Generally, bitter amino acids included L-tyrosine, L-isoleucine, L-leucine, L-phenylalanine, and L-valine. Sweet amino acids included L-serine, glycine, L-alanine, L-threonine, and L-proline. Umami amino acids included L-theanine, glutamic acid, and L-aspartic acid. The composition and content of amino acids as well as their degradation products and transformation products will affect the quality. According to research reports, organic acids are also key components in determining the taste and flavor, and play a crucial role in maintaining quality and nutritional value. Some organic acids, such as succinic acids, could protect mushrooms against various diseases due to their antioxidant activity and the organic acids in morels would have a positive effect on preservation (31). Therefore, both amino acids and organic acids can be important indexes to evaluate the quality and flavor of morels. In this study, three types of amino acids (N-oleoyl-L-serine, N-linoleoyl valine, and N-oleyl-isoleucine) and a total of 9 organic acids (citric acid, 2-furoic acid, 2-hydroxyhexadecanoic acid, 9-hydroxydecanoic acid, 2-isopropylmalic acid, 12-hydroxyheptadecanoic acid, 3-hydroxyanthranilic acid, dodecylbenzenesulfonic acid, and pyroglutamic acid) were identified as the differential metabolites. All these 12 metabolites showed similar trends that were significantly up-regulated in Guizhou morels relative to those from the other regions, responsible for the typical flavor of Guizhou morels. It has been reported that the contents of amino acids in edible fungi can be affected by many factors such as the cultivation mode, the climatic and geographical conditions, especially the temperature (32). Meanwhile, organic acids are mainly produced through the tricarboxylic acid or Krebs cycle, and many studies verified that a rising temperature increases the metabolic rate, hence decreasing the content of organic acids. In our study, the amino acids and organic acids contents of morels were positively correlated with the climate and temperature. As Guizhou has typical subtropical monsoon climate, the average temperature is relatively stable and higher than other regions, the precipitation and humidity suitable for morels growth interpreting the more significant amino acid and organic acids metabolism in Guizhou than the other three geographical origins. Overall, despite the regulation differences among regions, all the 32 differential metabolites including lipids, organic acids and amino acids proved to be efficient biomarkers for the discrimination of the regions and understanding of the metabolism signature effectively. Nonetheless, the strength of the results presented in this study is limited. More studies under controlled conditions are necessary to dissect the effect of environmental factors, intrinsic factors (life cycle and phylogeny) and cultivation/processing protocols on the physiochemical variation of morels, particularly in terms of lipids, amino acids, organic acids, and their detailed compositions. And then to assess the impact on potential applications. Moreover, although this metabolomics profiling approach can deliver noticeable trends of variation between different origins, suggesting the validity of this methodology. Further researches are required such as the absolute quantitation approach and the GC/MS-based platform study, which can provide insights into effect of origins on quality and flavor of morel samples.

## Conclusion

To the best of our knowledge, this is the first study focusing on morel samples derived from different geographical origins in China using UPLC-Q-TOF-MS-based untargeted metabolomics. PCA and OPLS-DA models were successfully applied to assess metabolites to differentiate the geographical origin of the samples. KEGG database analysis was also performed to identify the metabolic pathways associated with differential metabolites. As for the metabolomics profiles, the morels from Gansu, Guizhou, Liaoning, and Henan province displayed distinct clustering patterns, with 32 differentially expressed metabolites consisting of organic acids, amino acids, lipids, and ketones clearly classifying samples from different areas. Specifically, organic acids and amino acids were more prominent in Guizhou than other regions, while most lipids were scarce in Guizhou morels. The KEGG metabolic pathway analysis showed that lipid metabolism, amino acid metabolism and protein metabolism were closely related with geographical origins. In summary, this study yielded insights into the metabolism of morels, and presented a method to identify their geographical origin that may be used to develop a verifiable traceability system. It should be noted that the non-targeted metabolomics in the current study has sample distribution limitations. Hence diverse technologies and analytical methods of large samples would be required to verify data consistency and confirm the reliability of the potential biomarkers.

## Data Availability Statement

The original contributions presented in the study are included in the article/Supplementary Material, further inquiries can be directed to the corresponding author/s.

## Author Contributions

HD performed the experiment, generated the data, and wrote the original draft. CZ and XZ conceived and designed the experiments. HL wrote some parts of the article and improved the article further. MC provided the editorial support. HE and YZ helped performing the experiment and analyzed the data. HG reduced the repetition rate and polished the grammar. All authors contributed to the article and approved the manuscript.

## Funding

This work was based on research supported by the National Risk Assessment of Agro-Food Quality and Safety (GJFP20210504).

## Conflict of Interest

The authors declare that the research was conducted in the absence of any commercial or financial relationships that could be construed as a potential conflict of interest.

## Publisher's Note

All claims expressed in this article are solely those of the authors and do not necessarily represent those of their affiliated organizations, or those of the publisher, the editors and the reviewers. Any product that may be evaluated in this article, or claim that may be made by its manufacturer, is not guaranteed or endorsed by the publisher.
