# Supplementary material for: Metabolomics Analysis of Morchella sp. From Different Geographical Origins of China Using UPLC-Q-TOF-MS
Source: Front Nutr. 2022 Apr 5;9:865531. doi: 10.3389/fnut.2022.865531 (PMC9016275; doi:10.3389/fnut.2022.865531)
Supplement: Supplementary file 1 [file Table_1.docx]

Table S1. The sequences of *Morchella* *sp.*

| No. | Region | Species | Percent identity | Accession |
| --- | --- | --- | --- | --- |
| 1 | Gansu | *Morchella sextelata* | 99.57% | OM948735 |
| 2 |  | *Morchella sextelata* | 100% | OM961142 |
| 3 |  | *Morchella sextelata* | 99.71% | OM948783 |
| 4 |  | *Morchella sextelata* | 100% | OM948784 |
| 5 |  | *Morchella sextelata* | 99.86% | OM956125 |
| 6 |  | *Morchella sextelata* | 99.86% | OM956126 |
| 7 |  | *Morchella sextelata* | 99.72% | OM956128 |
| 8 |  | *Morchella sextelata* | 99.57% | OM956129 |
| 9 |  | *Morchella sextelata* | 99.71% | OM961394 |
| 10 |  | *Morchella sextelata* | 98.98% | OM956135 |
| 11 |  | *Morchella sextelata* | 98.09% | OM956131 |
| 12 |  | *Morchella sextelata* | 99.71% | OM962795 |
| 13 |  | *Morchella sextelata* | 99.57% | OM962795 |
| 14 |  | *Morchella sextelata* | 99.86% | OM956133 |
| 15 |  | *Morchella sextelata* | 99.72% | OM956134 |
| 16 | Henan | *Morchella sextelata* | 99.72% | OM956136 |
| 17 |  | *Morchella sextelata* | 100% | OM967216 |
| 18 |  | *Morchella sextelata* | 100% | OM967217 |
| 19 |  | *Morchella sextelata* | 99.71% | OM956137 |
| 20 |  | *Morchella sextelata* | 99.57% | OM956138 |
| 21 |  | *Morchella sextelata* | 99.86% | OM956139 |
| 22 |  | *Morchella sextelata* | 99.57% | OM956354 |
| 23 |  | *Morchella sextelata* | 99.86% | OM956357 |
| 24 |  | *Morchella sextelata* | 99.72% | OM956359 |
| 25 |  | *Morchella sextelata* | 99.86% | OM956364 |
| 26 |  | *Morchella sextelata* | 99.86% | OM956366 |
| 27 |  | *Morchella sextelata* | 99.57% | OM956369 |
| 28 |  | *Morchella sextelata* | 99.71% | OM956395 |
| 29 |  | *Morchella sextelata* | 100% | OM956804 |
| 30 |  | *Morchella sextelata* | 99.86% | OM956805 |
| 31 | Guizhou | *Morchella sextelata* | 99.86% | OM956824 |
| 32 |  | *Morchella sextelata* | 100% | OM956824 |
| 33 |  | *Morchella sextelata* | 99.29% | OM956831 |
| 34 |  | *Morchella sextelata* | 99.57% | OM956833 |
| 35 |  | *Morchella sextelata* | 97.65% | OM957498 |
| 36 |  | *Morchella sextelata* | 99.43% | OM957499 |
| 37 |  | *Morchella sextelata* | 98.58% | OM957536 |
| 38 |  | *Morchella sextelata* | 99.43% | OM957546 |
| 39 |  | *Morchella sextelata* | 100% | OM959221 |
| 40 |  | *Morchella sextelata* | 99.85% | OM959241 |
| 41 |  | *Morchella sextelata* | 99.28% | OM959237 |
| 42 |  | *Morchella sextelata* | 99.86% | OM962797 |
| 43 |  | *Morchella sextelata* | 99.71% | OM959621 |
| 44 |  | *Morchella sextelata* | 99.29% | OM959652 |
| 45 |  | *Morchella sextelata* | 99.14% | OM959658 |
| 46 | Liaoning | *Morchella sextelata* | 99.71% | OM959659 |
| 47 |  | *Morchella sextelata* | 99.72% | OM960562 |
| 48 |  | *Morchella sextelata* | 100% | OM960561 |
| 49 |  | *Morchella sextelata* | 99.71% | OM960631 |
| 50 |  | *Morchella sextelata* | 100% | OM960958 |
| 51 |  | *Morchella sextelata* | 100% | OM960962 |
| 52 |  | *Morchella sextelata* | 99.86% | OM962851 |
| 53 |  | *Morchella sextelata* | 99.57% | OM960968 |
| 54 |  | *Morchella sextelata* | 97.98% | OM960969 |
| 55 |  | *Morchella sextelata* | 97.43% | OM961045 |
| 56 |  | *Morchella sextelata* | 99.57% | OM961049 |
| 57 |  | *Morchella sextelata* | 99.71% | OM961048 |
| 58 |  | *Morchella sextelata* | 98.27% | OM964843 |
| 59 |  | *Morchella sextelata* | 99.43% | OM961179 |
| 60 |  | *Morchella sextelata* | 99.85% | OM961176 |
