# Supplementary material for: Metabolomics Analysis of Morchella sp. From Different Geographical Origins of China Using UPLC-Q-TOF-MS
Source: Front Nutr. 2022 Apr 5;9:865531. doi: 10.3389/fnut.2022.865531 (PMC9016275; doi:10.3389/fnut.2022.865531)
Supplement: Supplementary file 2 [file Table_2.docx]

**Table S2|** Differentiating metabolites with pairwise between four regions with VIP > 1 and *P*-value < 0.05

| **No.** | **Mode** | **Metabolite** | **m/z** | **Retention time(s)** | **Formula** | **VIP** | **Fold change** | ***P*-value** | | |
| --- | --- | --- | --- | --- | --- | --- | --- | --- | --- | --- |
| Henan versus Guizhou | | | | | | | | | |  |
| 1 | neg | PE(18:2/0:0) | 476.2779 | 7.33335 | C23H44NO7P | 6.970851 | 3.898707 | 1.95E-06 | | |
| 2 | neg | LysoPC(18:1(9Z)) | 566.3465 | 8.743617 | C26H52NO7P | 6.890826 | 5.92695 | 1.43E-06 | | |
| 3 | neg | 1-(9Z,12Z-Octadecadienoyl-2-hydroxy-sn-glycero-3-phosphocholine | 564.3308 | 7.140033 | C26H50NO7P | 5.963081 | 4.231249 | 6.35E-07 | | |
| 4 | neg | LPE(18:2) | 476.2779 | 7.082033 | C23H44NO7P | 3.773394 | 4.051591 | 6.45E-06 | | |
| 5 | neg | Citric acid | 191.0198 | 1.072317 | C6H8O7 | 5.169571 | 0.584352 | 3.56E-07 | | |
| 6 | neg | PE(18:1(9Z)/0:0) | 478.2938 | 8.64645 | C23H46NO7P | 4.093172 | 3.623367 | 3.53E-06 | | |
| 7 | neg | PS(18:1(9Z)/0:0) | 522.283 | 8.85945 | C24H46NO9P | 2.498453 | 2.085062 | 0.000603 | | |
| 8 | neg | LysoPC(18:0) | 568.3618 | 10.91902 | C26H54NO7P | 2.078876 | 6.152872 | 1.23E-05 | | |
| 9 | neg | LysoPC(20:1(11Z)) | 594.3772 | 11.33133 | C28H56NO7P | 1.13757 | 13.8761 | 1.12E-06 | | |
| 10 | neg | 2-hydroxyhexadecanoic acid | 271.2282 | 12.48645 | C16H32O3 | 3.497118 | 0.25662 | 3.95E-05 | | |
| 11 | neg | LPC(18:3) | 562.3156 | 6.609717 | C26H48NO7P | 1.647806 | 5.117386 | 0.001156 | | |
| 12 | neg | LysoPE(0:0/18:1(11Z)) | 957.5967 | 8.64645 | C23H46NO7P | 1.162391 | 524.3723 | 4.71E-05 | | |
| 13 | neg | 9-Hydroxydecanoic acid | 187.134 | 5.496283 | C10H20O3 | 1.719315 | 0.100636 | 0.000779 | | |
| 14 | neg | 2-Isopropylmalic acid | 175.0613 | 3.056717 | C7H12O5 | 1.691072 | 0.332236 | 6.64E-08 | | |
| 15 | neg | Mannitol | 181.0718 | 0.761833 | C6H14O6 | 2.049261 | 0.701299 | 0.00581 | | |
| 16 | neg | Uridine 5'-Diphosphogalactose | 565.0475 | 0.995483 | C15H24N2O17P2 | 1.806032 | 9.47404 | 6.86E-05 | | |
| 17 | neg | Malic acid | 133.0141 | 0.83965 | C4H6O5 | 1.670083 | 0.712866 | 0.004186 | | |
| 18 | neg | 12-hydroxyheptadecanoic acid | 285.2437 | 12.50645 | C17H34O3 | 1.474766 | 0.065908 | 0.009222 | | |
| 19 | neg | 3-Hydroxyanthranilic acid | 152.0352 | 3.097217 | C7H7NO3 | 1.694438 | 0.396634 | 4.41E-06 | | |
| 20 | neg | 9Z,12Z-Linoleic acid | 559.4731 | 13.66755 | C18H32O2 | 8.788556 | 0.619593 | 7.46E-07 | | |
| 21 | neg | LysoPC(20:2(11Z,14Z)) | 592.3619 | 9.303433 | C28H54NO7P | 1.037692 | 7.235214 | 8.44E-06 | | |
| 22 | pos | Farnesyl acetone | 263.2365 | 12.24783 | C18H30O | 2.559786 | 1.706796 | 0.000126 | | |
| 23 | pos | Tetrahydrodeoxycortisol | 368.279 | 9.489133 | C21H34O4 | 2.028486 | 0.363121 | 7E-08 | | |
| 24 | pos | 2-Methylbutyroylcarnitine | 246.1699 | 3.187433 | C12H23NO4 | 2.01618 | 2.325142 | 9.66E-05 | | |
| 25 | pos | Sphingosine | 300.2892 | 11.71353 | C18H37NO2 | 2.569239 | 0.376514 | 0.000352 | | |
| 26 | pos | N-Oleoyl-Phenylalanine | 430.331 | 14.89505 | C27H43NO3 | 1.934922 | 0.667629 | 0.009195 | | |
| 27 | neg | N-Oleoyl-L-Serine | 368.2803 | 11.50683 | C21H39NO4 | 1.234927 | 0.300516 | 1.61E-07 | | |
| 28 | pos | Oleoyl Ethanolamide | 326.3049 | 12.33233 | C20H39NO2 | 2.353241 | 0.513027 | 0.0005 | | |
| 29 | pos | (13R,14R)-7-Labdene-13,14,15-triol | 366.2996 | 12.1655 | C20H36O3 | 1.613173 | 0.503064 | 6.2E-05 | | |
| 30 | neg | Gluconic acid | 195.051 | 0.781167 | C6H12O7 | 2.909962 | 0.434568 | 6.39E-06 | | |
| 31 | pos | MG(0:0/18:1(9Z)/0:0) | 357.2992 | 13.68177 | C21H40O4 | 1.05445 | 3.382313 | 0.000449 | | |
| 32 | pos | 2-Linoleoyl Glycerol | 355.2835 | 12.24783 | C21H38O4 | 3.901264 | 1.739285 | 0.000203 | | |
| 33 | pos | MG(18:1(9Z)/0:0/0:0)[rac] | 357.3002 | 14.00458 | C21H40O4 | 2.481919 | 1.983785 | 0.001477 | | |
| 34 | pos | Linoleoyl Ethanolamide | 324.2892 | 10.2546 | C20H37NO2 | 5.571841 | 0.459836 | 0.00047 | | |
| 35 | neg | 2-Furoic acid | 111.0085 | 1.072317 | C5H4O3 | 1.921385 | 0.563896 | 5.96E-08 | | |
| 36 | pos | Ganoderic acid alpha | 616.3426 | 7.617733 | C32H46O9 | 2.014569 | 8.946227 | 0.000269 | | |
| 37 | pos | N-linoleoyl valine | 380.3154 | 12.86563 | C23H41NO3 | 4.241057 | 0.649084 | 0.001063 | | |
| 38 | pos | Arachidonoyl Serinol | 395.3264 | 6.489267 | C23H39NO3 | 1.040746 | 0.226991 | 0.007379 | | |
| 39 | pos | Vaccenic acid | 283.2627 | 15.12253 | C18H34O2 | 1.532226 | 1.20555 | 0.006098 | | |
| 40 | neg | LysoPA(0:0/18:2(9Z,12Z)) | 433.2358 | 13.68688 | C21H39O7P | 3.107524 | 4.724571 | 8.67E-07 | | |
| 41 | neg | LysoPC(16:0) | 540.3305 | 7.833 | C24H50NO7P | 1.60656 | 6.473461 | 3.51E-05 | | |
| 42 | pos | (3beta,22E,24R)-Ergosta-4,6,8(14),22-tetraen-3-ol | 377.3197 | 13.1213 | C28H42O | 2.176353 | 1.356555 | 0.000224 | | |
| 43 | pos | 3-Methyl-5-pentyl-2-furannonanoic acid | 309.2418 | 13.10097 | C19H32O3 | 2.063944 | 0.292681 | 0.000424 | | |
| 44 | pos | Linoleamide | 559.5183 | 11.37505 | C18H33NO | 2.481669 | 0.349411 | 0.001511 | | |
| 45 | neg | N-Oleyl-Isoleucine | 394.3324 | 14.942 | C24H45NO3 | 3.218482 | 0.573401 | 0.000871 | | |
| 46 | pos | 2-Formaminobenzoylacetate | 208.0606 | 1.100867 | C10H9NO4 | 1.154251 | 3.519773 | 2.77E-06 | | |
| 47 | pos | 1-Stearoyl-sn-glycero-3-phosphocholine | 524.37 | 10.89623 | C26H54NO7P | 3.959997 | 13.72693 | 1.34E-06 | | |
| 48 | neg | 1-Palmitoyl Lysophosphatidic Acid | 409.2358 | 13.68688 | C19H39O7P | 3.424535 | 3.439765 | 2.65E-06 | | |
| 49 | pos | PC(16:1(9E)/0:0) | 494.3235 | 6.905583 | C24H48NO7P | 3.185734 | 11.32803 | 1.34E-06 | | |
| 50 | pos | PE(16:0/0:0) | 454.2923 | 8.162367 | C21H44NO7P | 5.746396 | 7.895429 | 1.36E-07 | | |
| 51 | pos | LysoPE(16:1(9Z)/0:0) | 452.2768 | 6.866583 | C21H42NO7P | 1.358456 | 2.634676 | 0.000363 | | |
| 52 | pos | 1-palmitoyl-2-hydroxy-sn-glycero-3-phosphoethanolamine | 454.2921 | 7.772883 | C21H44NO7P | 2.05861 | 7.085745 | 1.59E-07 | | |
| 53 | neg | Dopaquinone | 176.0354 | 2.459083 | C9H9NO4 | 1.104733 | 0.585992 | 0.022875 | | |
| 54 | neg | Citramalic acid | 129.0191 | 1.091483 | C5H8O5 | 1.043346 | 0.487464 | 1.47E-09 | | |
| 55 | neg | Pyroglutamic acid | 128.035 | 1.091483 | C5H7NO3 | 1.890093 | 0.333157 | 1.21E-05 | | |
| 56 | neg | (R)-2-hydroxystearic acid | 299.2593 | 14.90367 | C18H36O3 | 1.705719 | 0.278118 | 0.001623 | | |
| 57 | pos | Pc(16:0/0:0) | 496.3388 | 7.831717 | C24H50NO7P | 3.575138 | 9.79105 | 1.61E-06 | | |
| 58 | neg | 12-OAHSA | 563.5042 | 15.11415 | C36H68O4 | 2.411203 | 0.791912 | 0.017505 | | |
| 59 | pos | PS(18:2(9Z,12Z)/20:1(11Z)) | 814.5573 | 11.31555 | C44H80NO10P | 1.18067 | 16.98503 | 2.53E-07 | | |
| 60 | pos | 1-Oleoyl Lysophosphatidic Acid (sodium salt) | 437.264 | 11.77503 | C21H41O7P | 2.220989 | 18.02467 | 2.32E-06 | | |
| 61 | pos | 5'-S-methyl-5'-thioadenosine | 298.0967 | 2.699783 | C11H15N5O3S | 1.165871 | 2.410496 | 0.011497 | | |
| 62 | neg | Pc(16:0/12-Hete) | 842.5562 | 15.73445 | C44H80NO9P | 1.170358 | 7.026762 | 3.68E-06 | | |
| 63 | neg | Reduced haloperidol | 398.1317 | 13.85955 | C21H25ClFNO2 | 1.633132 | 0.631385 | 7E-07 | | |
| 64 | neg | Dodecylbenzenesulfonic acid | 325.1844 | 15.13332 | C18H30O3S | 1.54922 | 0.567115 | 5.82E-09 | | |
| Gansu versus Guizhou | | | | | | | | | | |
| 1 | neg | PE(18:2/0:0) | 476.2779 | 7.33335 | C23H44NO7P | 5.551718 | 3.274151 | 0.000714 | | |
| 2 | neg | LysoPC(18:1(9Z)) | 566.3465 | 8.743617 | C26H52NO7P | 5.264437 | 3.953845 | 5.7E-05 | | |
| 3 | neg | 1-(9Z,12Z-Octadecadienoyl-2-hydroxy-sn-glycero-3-phosphocholine | 564.3308 | 7.140033 | C26H50NO7P | 4.991211 | 3.299396 | 3.33E-05 | | |
| 4 | neg | Cytochalasin Opho | 496.2676 | 8.373133 | C28H37NO4 | 3.387354 | 1.548515 | 0.030681 | | |
| 5 | neg | Cyclohexaneacetic acid | 343.2124 | 6.2489 | C8H14O2 | 1.085236 | 0.221223 | 0.045128 | | |
| 6 | neg | LPE(18:2) | 476.2779 | 7.082033 | C23H44NO7P | 2.986386 | 3.303349 | 0.001932 | | |
| 7 | neg | Citric acid | 191.0198 | 1.072317 | C6H8O7 | 3.637973 | 0.756046 | 0.001828 | | |
| 8 | neg | PE(18:1(9Z)/0:0) | 478.2938 | 8.64645 | C23H46NO7P | 2.709926 | 2.438336 | 0.00142 | | |
| 9 | neg | PS(18:1(9Z)/0:0) | 522.283 | 8.85945 | C24H46NO9P | 1.814117 | 1.665646 | 0.006804 | | |
| 10 | neg | LysoPC(18:0) | 568.3618 | 10.91902 | C26H54NO7P | 2.264704 | 6.233484 | 4.88E-06 | | |
| 11 | neg | (±)9-HpODE | 311.2227 | 6.589883 | C18H32O4 | 2.82657 | 0.493729 | 0.007786 | | |
| 12 | neg | LysoPC(20:1(11Z)) | 594.3772 | 11.33133 | C28H56NO7P | 1.01162 | 11.69572 | 5.64E-05 | | |
| 13 | neg | 9-HOTE | 293.2126 | 8.898117 | C18H30O3 | 3.333901 | 0.496768 | 0.001782 | | |
| 14 | neg | 2-hydroxyhexadecanoic acid | 271.2282 | 12.48645 | C16H32O3 | 4.226333 | 0.195563 | 6.4E-06 | | |
| 15 | neg | 9-hydroxy-10,12-octadecadienoic acid | 591.463 | 7.928817 | C18H32O3 | 2.111464 | 0.657849 | 0.00438 | | |
| 16 | neg | 9-Hydroxydecanoic acid | 187.134 | 5.496283 | C10H20O3 | 2.060128 | 0.04631 | 0.000404 | | |
| 17 | neg | 2-Isopropylmalic acid | 175.0613 | 3.056717 | C7H12O5 | 1.720156 | 0.386168 | 1.49E-06 | | |
| 18 | neg | Uridine 5'-Diphosphogalactose | 565.0475 | 0.995483 | C15H24N2O17P2 | 1.845302 | 8.345789 | 4.8E-07 | | |
| 19 | neg | 12-hydroxyheptadecanoic acid | 285.2437 | 12.50645 | C17H34O3 | 1.7729 | 0.047487 | 0.008035 | | |
| 20 | pos | N-(1-Deoxy-1-fructosyl)leucine | 276.1439 | 1.298183 | C12H23NO7 | 3.527693 | 4.040491 | 0.001883 | | |
| 21 | neg | 3-Hydroxyanthranilic acid | 152.0352 | 3.097217 | C7H7NO3 | 1.662341 | 0.469284 | 1.55E-05 | | |
| 22 | neg | FA(18:1(OH3)) | 329.2335 | 4.936967 | C18H34O5 | 4.98881 | 0.43136 | 0.000317 | | |
| 23 | neg | Ajmaline | 325.1942 | 5.683433 | C20H26N2O2 | 1.809353 | 0.557704 | 0.005108 | | |
| 24 | pos | Farnesyl acetone | 263.2365 | 12.24783 | C18H30O | 2.003503 | 1.428584 | 0.000358 | | |
| 25 | pos | (±)-(Z)-2-(5-Tetradecenyl)cyclobutanone | 282.2787 | 13.2858 | C18H32O | 2.557769 | 0.547096 | 0.009824 | | |
| 26 | pos | Tetrahydrodeoxycortisol | 368.279 | 9.489133 | C21H34O4 | 2.367229 | 0.302417 | 2.1E-08 | | |
| 27 | pos | 2-Methylbutyroylcarnitine | 246.1699 | 3.187433 | C12H23NO4 | 2.323576 | 2.303988 | 1.56E-05 | | |
| 28 | pos | Amprotropine | 308.2216 | 4.4817 | C18H29NO3 | 2.430165 | 0.310331 | 0.02285 | | |
| 29 | pos | Sphingosine | 300.2892 | 11.71353 | C18H37NO2 | 3.064909 | 0.324494 | 3.73E-05 | | |
| 30 | pos | N-Oleoyl-Phenylalanine | 430.331 | 14.89505 | C27H43NO3 | 3.011106 | 0.467507 | 1.92E-05 | | |
| 31 | neg | N-Oleoyl-L-Serine | 368.2803 | 11.50683 | C21H39NO4 | 1.37129 | 0.256311 | 1.02E-07 | | |
| 32 | pos | Oleoyl Ethanolamide | 326.3049 | 12.33233 | C20H39NO2 | 3.010994 | 0.441084 | 8.71E-07 | | |
| 33 | pos | (13R,14R)-7-Labdene-13,14,15-triol | 366.2996 | 12.1655 | C20H36O3 | 1.713974 | 0.472488 | 8.5E-05 | | |
| 34 | pos | Phytosphingosine | 318.3 | 6.531117 | C18H39NO3 | 1.245302 | 0.294215 | 0.000169 | | |
| 35 | pos | Sphingofungin A | 432.3108 | 15.08137 | C21H41N3O6 | 2.078502 | 9.113391 | 0.030393 | | |
| 36 | pos | 5-Hydroxyindol-2-carboxylic acid | 178.0494 | 2.461967 | C9H7NO3 | 1.367732 | 2.866822 | 0.045656 | | |
| 37 | pos | 2-Linoleoyl Glycerol | 355.2835 | 12.24783 | C21H38O4 | 2.967646 | 1.433712 | 0.000549 | | |
| 38 | pos | MG(18:1(9Z)/0:0/0:0)[rac] | 357.3002 | 14.00458 | C21H40O4 | 1.218437 | 1.297438 | 0.020489 | | |
| 39 | pos | Linoleoyl Ethanolamide | 324.2892 | 10.2546 | C20H37NO2 | 5.868052 | 0.515496 | 0.000322 | | |
| 40 | neg | 2-Furoic acid | 111.0085 | 1.072317 | C5H4O3 | 1.464106 | 0.716121 | 0.000319 | | |
| 41 | pos | N-(1-Deoxy-1-fructosyl)valine | 262.1283 | 0.8292 | C11H21NO7 | 1.654493 | 4.830365 | 0.00431 | | |
| 42 | pos | 7-Methylguanosine | 281.1131 | 2.461967 | C11H16N5O5+ | 1.151816 | 0.721197 | 0.000736 | | |
| 43 | pos | Ganoderic acid alpha | 616.3426 | 7.617733 | C32H46O9 | 2.29323 | 10.06997 | 0.000112 | | |
| 44 | pos | N-linoleoyl valine | 380.3154 | 12.86563 | C23H41NO3 | 4.013586 | 0.665426 | 0.010174 | | |
| 45 | pos | Vicenistatin | 501.3679 | 15.1847 | C30H48N2O4 | 7.019562 | 20.98901 | 0.011207 | | |
| 46 | neg | Prolylhydroxyproline | 227.1038 | 2.7654 | C10H16N2O4 | 1.023293 | 0.190692 | 1.62E-05 | | |
| 47 | pos | Arachidonoyl Serinol | 395.3264 | 6.489267 | C23H39NO3 | 1.281126 | 0.145813 | 0.003249 | | |
| 48 | neg | LysoPA(0:0/18:2(9Z,12Z)) | 433.2358 | 13.68688 | C21H39O7P | 4.973229 | 8.272379 | 8.2E-08 | | |
| 49 | neg | LysoPC(16:0) | 540.3305 | 7.833 | C24H50NO7P | 1.857299 | 7.009774 | 3.49E-05 | | |
| 50 | pos | Linoleamide | 559.5183 | 11.37505 | C18H33NO | 3.180016 | 0.278954 | 0.000475 | | |
| 51 | neg | N-Oleyl-Isoleucine | 394.3324 | 14.942 | C24H45NO3 | 3.007338 | 0.604177 | 0.002341 | | |
| 52 | neg | Hydronnonanedioate | 187.0978 | 4.033333 | C9H16O4 | 2.761551 | 0.318377 | 0.003934 | | |
| 53 | pos | 1-Stearoyl-sn-glycero-3-phosphocholine | 524.37 | 10.89623 | C26H54NO7P | 3.337948 | 10.10459 | 0.000119 | | |
| 54 | neg | 1-Palmitoyl Lysophosphatidic Acid | 409.2358 | 13.68688 | C19H39O7P | 5.759353 | 7.04195 | 3.71E-05 | | |
| 55 | pos | PC(16:1(9E)/0:0) | 494.3235 | 6.905583 | C24H48NO7P | 2.02384 | 5.997138 | 0.001164 | | |
| 56 | pos | Succinoadenosine | 384.1147 | 2.403467 | C14H17N5O8 | 3.313948 | 2.647058 | 0.043118 | | |
| 57 | pos | PE(16:0/0:0) | 454.2923 | 8.162367 | C21H44NO7P | 4.695666 | 6.388163 | 0.000129 | | |
| 58 | pos | 1-palmitoyl-2-hydroxy-sn-glycero-3-phosphoethanolamine | 454.2921 | 7.772883 | C21H44NO7P | 1.59153 | 5.359054 | 0.000313 | | |
| 59 | neg | Pyroglutamic acid | 128.035 | 1.091483 | C5H7NO3 | 1.999123 | 0.3362 | 1.29E-05 | | |
| 60 | neg | (R)-2-hydroxystearic acid | 299.2593 | 14.90367 | C18H36O3 | 2.203768 | 0.156196 | 0.000263 | | |
| 61 | pos | Pc(16:0/0:0) | 496.3388 | 7.831717 | C24H50NO7P | 3.026178 | 7.552795 | 0.000287 | | |
| 62 | pos | 1-Oleoyl Lysophosphatidic Acid (sodium salt) | 437.264 | 11.77503 | C21H41O7P | 2.265133 | 13.67809 | 2.31E-12 | | |
| 63 | neg | 12-Hydroxydodecanoic acid | 215.1655 | 7.1207 | C12H24O3 | 1.185983 | 0.018719 | 0.000986 | | |
| 64 | pos | DG(15:0/18:4(6Z,9Z,12Z,15Z)/0:0) | 575.4655 | 16.56313 | C36H62O5 | 1.521052 | 0.621079 | 0.025089 | | |
| 65 | neg | Dodecylbenzenesulfonic acid | 325.1844 | 15.13332 | C18H30O3S | 1.167021 | 0.712818 | 9.42E-05 | | |
| 66 | neg | Ac-Ser-Asp-Lys-Pro-OH | 508.2003 | 9.419417 | C20H33N5O9 | 1.203033 | 0.109222 | 6.36E-11 | | |
| Liaoning versus Guizhou | | | | | | | | |  |  |
| 1 | neg | PE(18:2/0:0) | 476.2779 | 7.33335 | C23H44NO7P | 5.655082 | 2.188269 | 0.01431 | | |
| 2 | neg | LysoPC(18:1(9Z)) | 566.3465 | 8.743617 | C26H52NO7P | 4.949854 | 2.623262 | 0.010073 | | |
| 3 | neg | 1-(9Z,12Z-Octadecadienoyl-2-hydroxy-sn-glycero-3-phosphocholine | 564.3308 | 7.140033 | C26H50NO7P | 4.534066 | 2.244361 | 0.01865 | | |
| 4 | neg | LPE(18:2) | 476.2779 | 7.082033 | C23H44NO7P | 2.489109 | 1.939393 | 0.034711 | | |
| 5 | neg | 1-a,24R,25-Trihydroxyvitamin D2 | 489.3212 | 12.7866 | C28H44O4 | 1.21068 | 1.424216 | 0.02191 | | |
| 6 | neg | Citric acid | 191.0198 | 1.072317 | C6H8O7 | 5.01691 | 0.772105 | 0.00564 | | |
| 7 | neg | PE(18:1(9Z)/0:0) | 478.2938 | 8.64645 | C23H46NO7P | 2.705326 | 1.863119 | 0.033915 | | |
| 8 | neg | LysoPC(18:0) | 568.3618 | 10.91902 | C26H54NO7P | 1.606717 | 2.673538 | 0.003362 | | |
| 9 | neg | 2-hydroxyhexadecanoic acid | 271.2282 | 12.48645 | C16H32O3 | 3.933976 | 0.543678 | 0.021191 | | |
| 10 | neg | D-Pantothenic Acid | 218.1035 | 2.359917 | C9H17NO5 | 1.012644 | 1.543197 | 0.007377 | | |
| 11 | neg | 9-Hydroxydecanoic acid | 187.134 | 5.496283 | C10H20O3 | 2.862632 | 0.17995 | 0.001963 | | |
| 12 | neg | 2-Isopropylmalic acid | 175.0613 | 3.056717 | C7H12O5 | 2.710544 | 0.368761 | 1.65E-06 | | |
| 13 | neg | 12-hydroxyheptadecanoic acid | 285.2437 | 12.50645 | C17H34O3 | 2.593167 | 0.127396 | 0.014613 | | |
| 14 | neg | (2S)-2-amino-3-(1H-indol-3-yl)propanoic acid | 203.0829 | 2.8249 | C11H12N2O2 | 1.430598 | 0.639547 | 0.005714 | | |
| 15 | neg | Gamma-Glutamylleucine | 241.1192 | 3.356383 | C11H20N2O5 | 1.080797 | 0.461272 | 0.000547 | | |
| 16 | pos | 2-Hydroxycinnamic acid | 182.0809 | 1.100867 | C9H8O3 | 1.44231 | 0.56296 | 0.004055 | | |
| 17 | neg | 3-Hydroxyanthranilic acid | 152.0352 | 3.097217 | C7H7NO3 | 3.018867 | 0.340817 | 1.46E-06 | | |
| 18 | neg | 9Z,12Z-Linoleic acid | 559.4731 | 13.66755 | C18H32O2 | 7.399635 | 0.823152 | 0.016868 | | |
| 19 | neg | Methylmalonic acid | 117.019 | 1.206967 | C4H6O4 | 1.123046 | 0.660715 | 0.00389 | | |
| 20 | pos | Farnesyl acetone | 263.2365 | 12.24783 | C18H30O | 3.215208 | 1.574305 | 0.006133 | | |
| 21 | pos | Tetrahydrodeoxycortisol | 368.279 | 9.489133 | C21H34O4 | 2.057366 | 0.602015 | 0.003864 | | |
| 22 | neg | N-Oleoyl-L-Serine | 368.2803 | 11.50683 | C21H39NO4 | 1.410196 | 0.522334 | 0.000956 | | |
| 23 | neg | Uridine monophosphate (UMP) | 323.0284 | 0.800667 | C9H13N2O9P | 1.176358 | 1.535035 | 0.028103 | | |
| 24 | pos | Betaine | 118.0856 | 0.809383 | C5H11NO2 | 1.960943 | 0.55603 | 0.003306 | | |
| 25 | pos | MG(0:0/18:1(9Z)/0:0) | 357.2992 | 13.68177 | C21H40O4 | 1.247737 | 2.792471 | 0.005029 | | |
| 26 | pos | 2-Linoleoyl Glycerol | 355.2835 | 12.24783 | C21H38O4 | 5.241038 | 1.657047 | 0.004847 | | |
| 27 | pos | MG(18:1(9Z)/0:0/0:0)[rac] | 357.3002 | 14.00458 | C21H40O4 | 2.82031 | 1.727462 | 0.015049 | | |
| 28 | neg | 2-Furoic acid | 111.0085 | 1.072317 | C5H4O3 | 1.680689 | 0.788422 | 0.007516 | | |
| 29 | neg | L(+)-Arginine | 173.1043 | 0.721833 | C6H14N4O2 | 1.286088 | 0.504713 | 0.00186 | | |
| 30 | pos | N-linoleoyl valine | 380.3154 | 12.86563 | C23H41NO3 | 4.947561 | 0.754654 | 0.016238 | | |
| 31 | neg | Prolylhydroxyproline | 227.1038 | 2.7654 | C10H16N2O4 | 1.248924 | 0.364684 | 0.001546 | | |
| 32 | neg | LysoPA(0:0/18:2(9Z,12Z)) | 433.2358 | 13.68688 | C21H39O7P | 2.992579 | 3.196847 | 0.025943 | | |
| 33 | neg | LysoPC(16:0) | 540.3305 | 7.833 | C24H50NO7P | 1.125154 | 2.754783 | 0.038865 | | |
| 34 | pos | (3beta,22E,24R)-Ergosta-4,6,8(14),22-tetraen-3-ol | 377.3197 | 13.1213 | C28H42O | 3.421351 | 1.361056 | 0.001165 | | |
| 35 | pos | 3-Methyl-5-pentyl-2-furannonanoic acid | 309.2418 | 13.10097 | C19H32O3 | 2.979698 | 0.297096 | 0.001655 | | |
| 36 | pos | Linoleamide | 559.5183 | 11.37505 | C18H33NO | 3.446586 | 0.514617 | 0.019905 | | |
| 37 | neg | N-Oleyl-Isoleucine | 394.3324 | 14.942 | C24H45NO3 | 3.700632 | 0.724945 | 0.026675 | | |
| 38 | pos | 1-Stearoyl-sn-glycero-3-phosphocholine | 524.37 | 10.89623 | C26H54NO7P | 2.698729 | 4.197005 | 0.00241 | | |
| 39 | neg | 1-Palmitoyl Lysophosphatidic Acid | 409.2358 | 13.68688 | C19H39O7P | 3.234239 | 2.326478 | 0.016035 | | |
| 40 | pos | PC(16:1(9E)/0:0) | 494.3235 | 6.905583 | C24H48NO7P | 1.914995 | 3.462787 | 0.017419 | | |
| 41 | pos | PE(16:0/0:0) | 454.2923 | 8.162367 | C21H44NO7P | 4.77785 | 3.808594 | 0.004558 | | |
| 42 | pos | 1-palmitoyl-2-hydroxy-sn-glycero-3-phosphoethanolamine | 454.2921 | 7.772883 | C21H44NO7P | 1.711559 | 3.578342 | 0.006679 | | |
| 43 | neg | Dopaquinone | 176.0354 | 2.459083 | C9H9NO4 | 1.534177 | 0.590303 | 0.036464 | | |
| 44 | neg | Citramalic acid | 129.0191 | 1.091483 | C5H8O5 | 1.170723 | 0.686959 | 9.06E-05 | | |
| 45 | neg | Pyroglutamic acid | 128.035 | 1.091483 | C5H7NO3 | 2.145495 | 0.54948 | 0.005345 | | |
| 46 | pos | Pc(16:0/0:0) | 496.3388 | 7.831717 | C24H50NO7P | 2.392681 | 3.352376 | 0.011335 | | |
| 47 | pos | 14-(hydroxymethyl)-5,9-dimethyltetracyclo[11.2.1.0 | 315.2314 | 14.98155 | C19H32O2 | 1.300981 | 1.250022 | 0.012627 | | |
| 48 | pos | 11Z-hexadecenoic acid | 237.2209 | 13.10097 | C16H30O2 | 1.010856 | 0.717177 | 0.035222 | | |
| 49 | neg | Indole-3-carboxyaldehyde | 144.0452 | 4.110667 | C9H7NO | 1.343576 | 0.315702 | 2.75E-05 | | |
| 50 | pos | 1-Oleoyl Lysophosphatidic Acid (sodium salt) | 437.264 | 11.77503 | C21H41O7P | 2.100021 | 8.653412 | 0.001746 | | |
| 51 | pos | MG(18:2(9Z,12Z)/0:0/0:0)[rac] | 355.2835 | 11.85753 | C21H38O4 | 1.12258 | 1.811061 | 0.00555 | | |
| 52 | pos | Adenylosuccinic acid | 464.0811 | 1.48335 | C14H18N5O11P | 1.387066 | 2.390243 | 0.016081 | | |
| 53 | neg | 12-Hydroxydodecanoic acid | 215.1655 | 7.1207 | C12H24O3 | 1.526578 | 0.204927 | 0.007378 | | |
| 54 | neg | Reduced haloperidol | 398.1317 | 13.85955 | C21H25ClFNO2 | 1.97021 | 0.739518 | 0.000325 | | |
| 55 | neg | Xanthurenic acid | 204.0303 | 2.707067 | C10H7NO4 | 1.389436 | 0.212458 | 3.97E-05 | | |
| 56 | neg | Dodecylbenzenesulfonic acid | 325.1844 | 15.13332 | C18H30O3S | 1.223002 | 0.829193 | 0.008691 | | |
| 57 | neg | Alpha-D-Glucose-1,6-diphosphate | 338.9885 | 1.014983 | C6H14O12P2 | 1.266989 | 13.50076 | 0.046462 | | |
| 58 | neg | 9-PAHSA | 537.489 | 15.05648 | C34H66O4 | 1.008307 | 0.7012 | 0.005177 | | |
