# Supplementary figures and images for: Metabolomics Analysis of Morchella sp. From Different Geographical Origins of China Using UPLC-Q-TOF-MS
Source: Front Nutr. 2022 Apr 5;9:865531. doi: 10.3389/fnut.2022.865531 (PMC9016275; doi:10.3389/fnut.2022.865531)

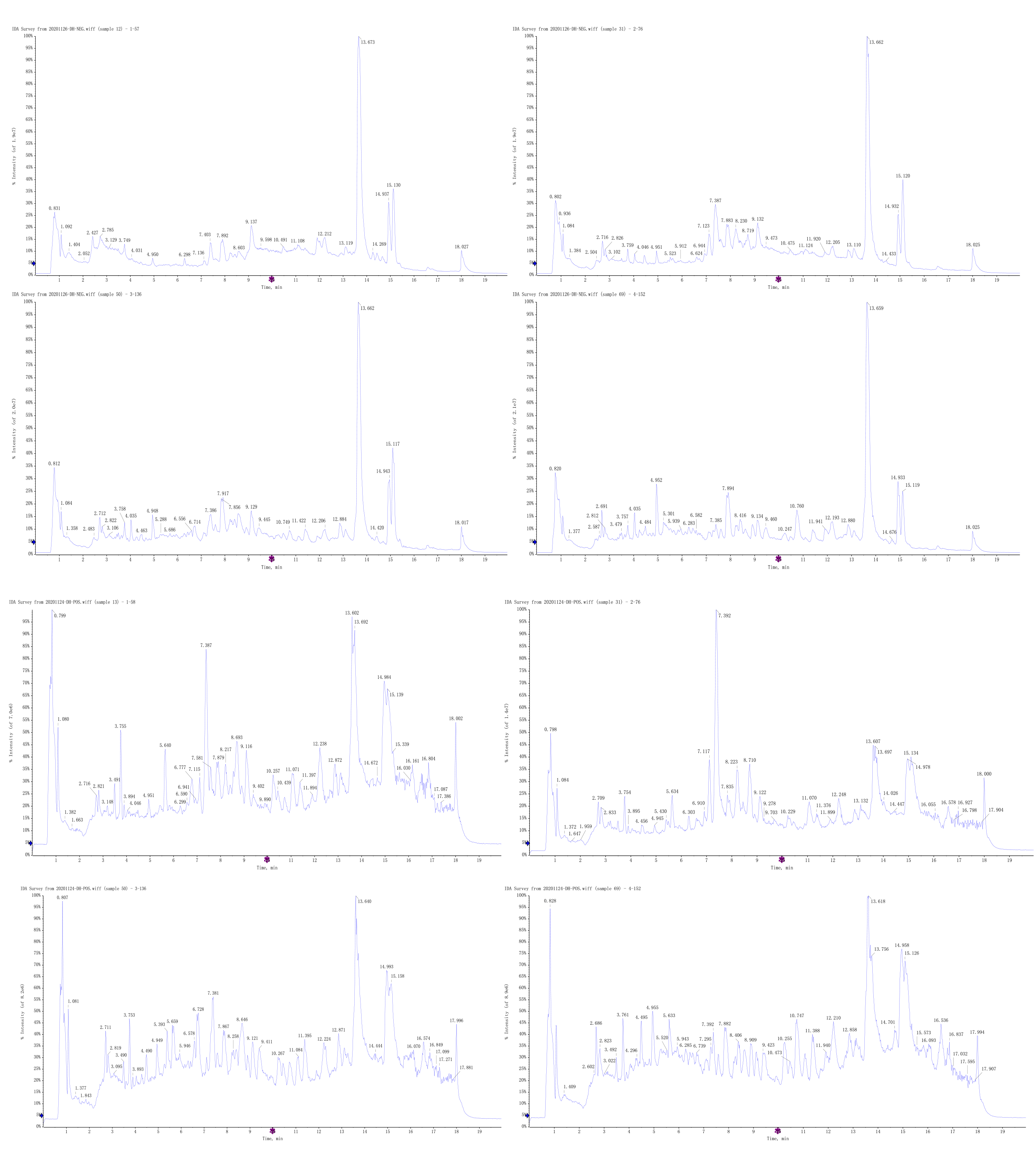

Supplement: Supplementary file 3 [file Image_1.tif]
